# A Centenarian

**Published:** 1888-10

**Authors:** 


### A Centenarian.

The hundredth birthday of Col. George L. Perkins was celebrated at Norwich, Conn., recently. The old gentleman looks as if he were good for another hundred, is actively engaged in business, walks four miles to his office every day, and is as erect and strong as any man of sixty. When his family physician died, a few years ago, he offered the vacant place to a young doctor, but with the remark that there was some risk in accepting it, as he had buried seven of his doctors already! He is full of reminiscences of the earlier years of the century, one of these being his ride on Robert Fulton's first steamboat on her trial trip. He was nineteen years younger than Napoleon, and was born in the same year with Byron.—*Philadelphia American.*

